# Supplementary figures and images for: Identifying patterns differing between high-dimensional datasets with generalized contrastive PCA
Source: PLoS Comput Biol. 2025 Feb 7;21(2):e1012747. doi: 10.1371/journal.pcbi.1012747 (PMC11841894; doi:10.1371/journal.pcbi.1012747)

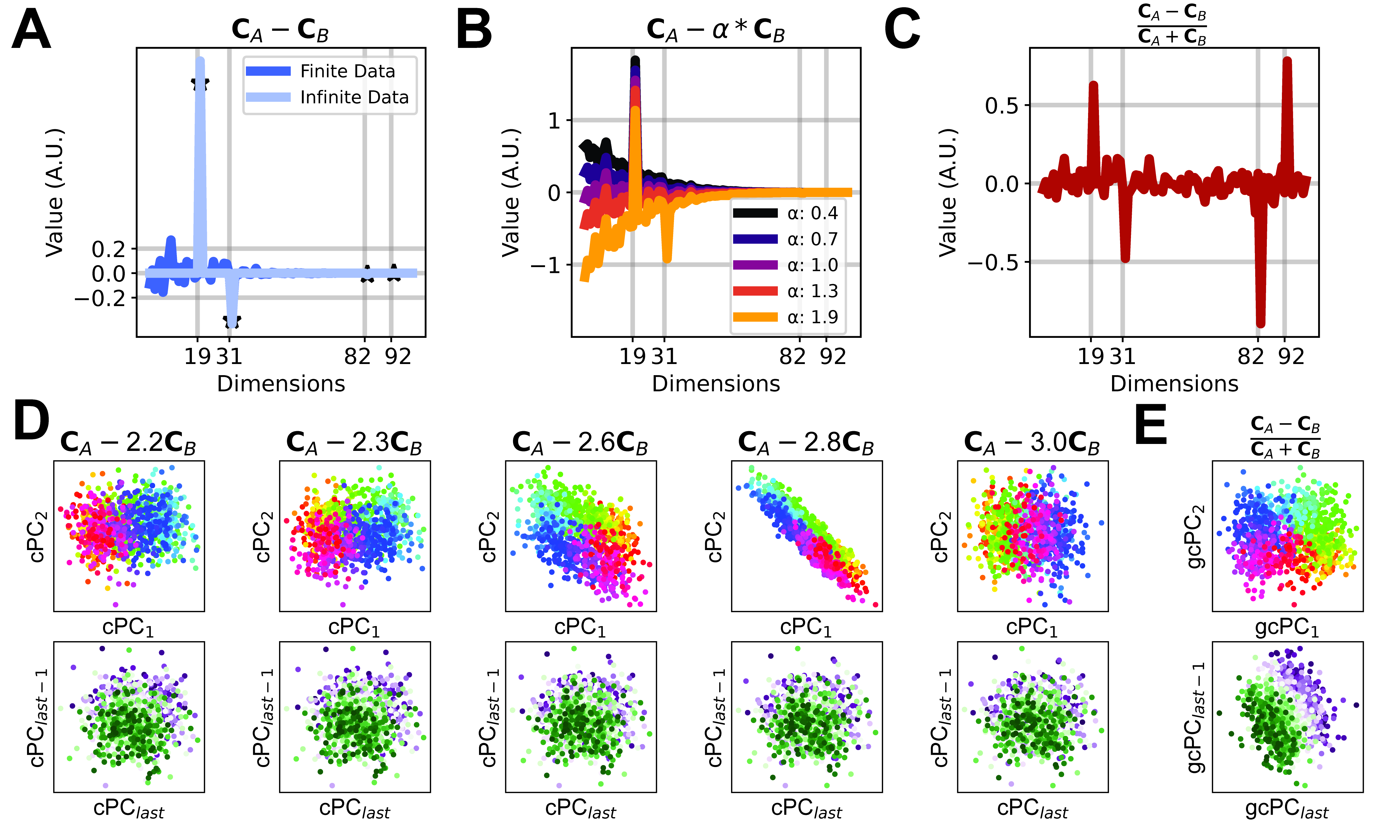

Supplement: S2Fig — (A) We generated synthetic data with enriched variance in both high and low variance dimensions. In condition A we enriched the variance in dimensions 19 and 92, and in condition B we enriched the variance in dimensions 31 and 82. This panel shows the finite and infinite data results for CA-CB. Stars represents the finite data value in the enriched dimensions. Even though the high variance dimensions are easy to detect with this method, the low variance ones are still occluded by spurious variability in high variance dimensions. (B) cPCA can reveal enriched high variance dimensions, but enriched low variance dimensions are hard to identify. (C) gcPCA can find all enriched dimensions simultaneously for conditions A and B. (D) The range of α values yielding the correct solution becomes narrow because the enriched dimensions have different absolute variance. (E) gcPCA correctly identifies all enriched dimensions in both conditions. (TIF) [file pcbi.1012747.s002.tif]

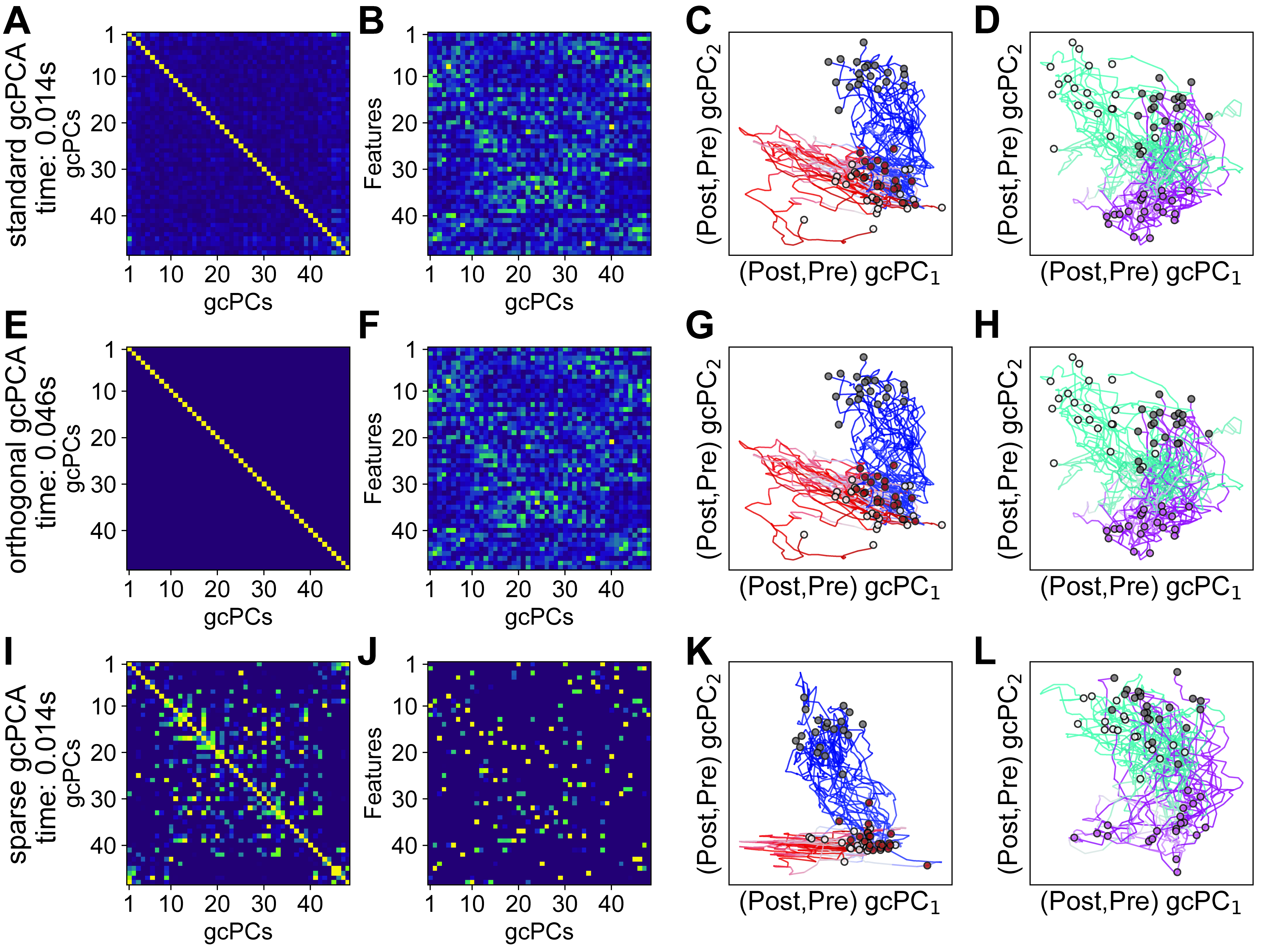

Supplement: S3Fig — (A) The gcPC covariance matrix for the hippocampal data in Fig 3 using gcPCA v4. The non-zero values off the diagonal reveal that, by default, gcPCs are not orthogonal in original feature space, though in practice they are usually quite close. (B) gcPC loadings for gcPCA v4. (C-D) The projection of task data onto the gcPCs exhibits spatial structure similar to PCs from the task data (Fig 3C). (E) Orthogonal gcPCA v4.1 finds gcPCs that are orthogonal in the original feature space, as shown by the absence of non-zero values off the diagonal. Note that the processing time is substantially longer. (F) For these datasets, the loadings for orthogonal gcPCA v4.1 are almost indistinguishable from standard gcPCA v4 (panel B). (G-H) Projecting task data onto the orthogonal gcPCs yields almost identical results to standard gcPCA v4 (panels C-D). (I) With sparse gcPCA, the sparsification of the gcPCs creates deviations from orthogonality. The processing time is also substantially longer. (J) Note that the loadings are sparser than in panels B and F, meaning that the most important features have been highlighted. (K-L) Task data projected onto the sparse gcPCs is somewhat distorted relative to panels C-D and G-H. (TIF) [file pcbi.1012747.s003.tif]
