# Supplementary material for: Identifying patterns differing between high-dimensional datasets with generalized contrastive PCA
Source: PLoS Comput Biol. 2025 Feb 7;21(2):e1012747. doi: 10.1371/journal.pcbi.1012747 (PMC11841894; doi:10.1371/journal.pcbi.1012747)
Supplement: Attachment — Submitted filename: gcPCA Review.pdf [file pcbi.1012747.s004.pdf]

de Oliveira et. al. proposed a new form of contrastive PCA that eliminates the need of a necessary hyperparameter. Contrastive PCA (cPCA) is a dimensionality reduction technique that specializes at discovering directions of variability that are more present in one dataset relative to the other (e.g. electrophysiological recordings during sleep before and after maze running). Ordinary PCA finds axes of maximal variability within one dataset by maximizing the quadratic form of the covariance matrix. The gist of contrastive PCA is to maximize the quadratic form that is the difference between the covariance matrix of dataset A vs dataset B. However, the caveat is that dimensions that have large variance in both datasets, which are not the focus of the comparative studies, can erroneously show up as the contrastive PC axes due to noise. To counter this bias, the previous Abid (2018) used a hyperparameter alpha to control how much covariance from the second condition to subtract from the first.

However, de Oliveira et. al. pointed out several drawbacks of this approach: 1) there is no principled way of selecting the hyperparameter; 2) the problem becomes asymmetrical; 3) dimensions with negative $x^T(C_A - \alpha C_B)x$ values are usually interpreted as enriched in dataset B, but that is not always true, as high variance dimensions are biased to have negative cPCA values when alpha is larger than 1. So their solution, named generalized contrastive PCA (gcPCA) is to find dimensions that maximize $\frac{x^T(C_A - C_B)x}{x^T(C_A + C_B)x}$. The reason why this formulation solves the problem is because large variance dimensions that are shared between A and B will be penalized through the denominator (however, this rationale was not explicitly explained in the paper in much detail).

The solution to gcPCA can be readily obtained by solving a generalized eigenvalue problem, although the dimensions are no longer orthogonal by default. They proposed a few variants of this model, including the ones with orthogonality or sparsity constraints. For the results, they first demonstrated through simulation why the hyperparameter was needed in the original cPCA, then how gcPCA could avoid it. They also showed that gcPCA can identify the axis that separates happy and sad faces in the Chicago face dataset. They also demonstrated how gcPCA discovers meaningful structure on a hippocampal electrophysiological recording as well as scRNA-seq data. Overall the idea is clever, the paper is clearly written, and the method has great potential.

But I have a few comments regarding the motivation for the objective function of the method, as well as the interpretations of the scientific findings.

- The reason why the gcPCA formulation of the objective function solves the problem was never explicitly explained in the paper. Although it might be obvious to the author, it would still be pedagogical to spell it out. (Similarly, the reason why alpha could solve the problem for cPCA was not fully explained either. Even though cPCA was not proposed by this paper, it would still be helpful for the reader to understand it more easily, especially since there’s a chapter in this paper devoted to demonstrating the utility of alpha).

- The application on the hippocampal dataset seems intriguing, but is way too vague as currently written. For instance, for terms like “replay of task-related spatial structure” (line 248), “structures of replay” (line 252) it is not clear what structure the authors were referring to. It could refer to the separation of the grey (stop) dots from the white (start) and red (air puff) in the top left subplot of Fig. 3D, which is indeed not present in the pre-task (top right subplot of Fig. 3D). But it could also refer to the separation of the blue and red lines, which does seem separated in the pre-task (top right of Fig. 3D) as well. What is the significance that start and air puff are overlapping in the danger run (top left of Fig. 3D), but separated in the safe run (bottom right of Fig. 3D)? What is the significance of the segregation of the blue (pre air-puff) and red lines (post air-puff)? Why are the start, non-airpuff and stop in the safe run separated in both pre-task and post-task subspaces?

- Additional controls are also needed to make sense of the results of Figure 3. What happens if one applies PCA to the Pre-Task data and visualizes task activity in this space? Similarly, what happens if one applies PCA to the Post-Task data and visualizes task activity in this space? Finally, If one applies PCA to the Task data directly what does the activity look like? To what extent is gcPCA essentially recovering the same PCs as these three simple analyses?

- In Figure 3 the authors mention they are using non-orthogonal gcPCA but they show the trajectories plotted against orthogonal axes. I assume that the authors are just orthogonalizing the pairs of dimensions, but it would be good to be explicit about this. In fact, it would be interesting to plot the non-orthogonal axes as arrows (i.e. point-to-tip visualization of a vector) in the low-D plots of Figure 3, or as a supplemental figure.

- Calling the spatial segregation in Fig. 3D “replay” requires a bit more explanation and justification. My understanding is that the top gcPC axes reflect directions of covariability enriched in post-task. Because these directions can distinguish spatially related firing during the task, this shows the enrichment of certain spatial information in the post-task. The interpretation of the result as “replay” should be better unpacked.

- The results on Figure 4 seem similarly short on details. However, I am not familiar with the scientific literature on ‘omics analysis to give detailed comments on this part of the manuscript.
