# Supplementary material for: Identifying patterns differing between high-dimensional datasets with generalized contrastive PCA
Source: PLoS Comput Biol. 2025 Feb 7;21(2):e1012747. doi: 10.1371/journal.pcbi.1012747 (PMC11841894; doi:10.1371/journal.pcbi.1012747)
Supplement: Attachment — Submitted filename: Response to Reviewers.pdf [file pcbi.1012747.s005.pdf]

Response to reviewers

We thank the reviewers for their thoughtful critiques, which have greatly strengthened the manuscript. The main changes we have made to address the reviewers' comments were expanding Figures 3 and 4 to include additional controls, as well as adding Fig. S1 (addressing the issue of LDA vs. gcPCA) and Fig. S3 (illustrating the effects of the orthogonality and sparsity constraints).

Our replies to the reviewers' comments are below in black text. Line numbers refer to the line numbers in the "track changes" version of the manuscript. Note: because of how the LaTeX document is organized, changes are only tracked in the main text and figures, and not in the references or supplementary figures.

Reviewer #1: de Oliveira et. al. proposed a new form of contrastive PCA that eliminates the need of a necessary hyperparameter. Contrastive PCA (cPCA) is a dimensionality reduction technique that specializes at discovering directions of variability that are more present in one dataset relative to the other (e.g. electrophysiological recordings during sleep before and after maze running). Ordinary PCA finds axes of maximal variability within one dataset by maximizing the quadratic form of the covariance matrix. The gist of contrastive PCA is to maximize the quadratic form that is the difference between the covariance matrix of dataset A vs dataset B. However, the caveat is that dimensions that have large variance in both datasets, which are not the focus of the comparative studies, can erroneously show up as the contrastive PC axes due to noise. To counter this bias, the previous Abid (2018) used a hyperparameter alpha to control how much covariance from the second condition to subtract from the first. However, de Oliveira et. al. pointed out several drawbacks of this approach: 1) there is no principled way of selecting the hyperparameter; 2) the problem becomes asymmetrical; 3) dimensions with negative values **EQUATION** are usually interpreted as enriched in dataset B, but that is not always true, as high variance dimensions are biased to have negative cPCA values when alpha is larger than 1. So their solution, named generalized contrastive PCA (gcPCA) is to find dimensions that maximize **EQUATION** . The reason why this formulation solves the problem is because large variance dimensions that are shared between A and B will be penalized through the denominator (however, this rationale was not explicitly explained in the paper in much detail). The solution to gcPCA can be readily obtained by solving a generalized eigenvalue problem, although the dimensions are no longer orthogonal by default. They proposed a few variants of this model, including the ones with orthogonality or sparsity constraints. For the results, they first demonstrated through simulation why the hyperparameter was needed in the original cPCA, then how gcPCA could avoid it. They also showed that gcPCA can identify the axis that separates happy and sad faces in the Chicago face dataset. They also demonstrated how gcPCA discovers meaningful structure on a hippocampal electrophysiological recording as well as scRNA-seq data. Overall the idea is clever, the paper is clearly written, and the method has great potential. But I have a few comments regarding the motivation for the objective function of the method, as well as the interpretations of the scientific findings.

- The reason why the gcPCA formulation of the objective function solves the problem was never explicitly explained in the paper. Although it might be obvious to the author, it would still be pedagogical to spell it out. (Similarly, the reason why alpha could solve the problem for cPCA was not fully explained either. Even though cPCA was not proposed by this paper, it would still be helpful for the reader to understand it more easily, especially since there's a chapter in this paper devoted to demonstrating the utility of alpha).

We have now updated the text to clarify these points (lines 128-130, 145-149, 154-158, 160-163, 206-208, 374-375, 392-395).

- The application on the hippocampal dataset seems intriguing, but is way too vague as currently written. For instance, for terms like "replay of task-related spatial structure" (line 248), "structures of replay" (line 252) it is not clear what structure the authors were referring to. It could refer to the separation of the grey (stop) dots from the white (start) and red (air puff) in the top left subplot of Fig. 3D, which is indeed not present in the pre-task (top right subplot of Fig. 3D). But it could also refer to the separation of the blue and red lines, which does seem separated in the pre-task (top right of Fig. 3D) as well.
- Additional controls are also needed to make sense of the results of Figure 3. What happens if one applies PCA to the Pre-Task data and visualizes task activity in this space? Similarly, what happens if one applies PCA to the Post-Task data and visualizes task activity in this space? Finally, If one applies PCA to the Task data directly what does the activity look like? To what extent is gcPCA essentially recovering the same PCs as these three simple analyses?
- Calling the spatial segregation in Fig. 3D "replay" requires a bit more explanation and justification. My understanding is that the top gcPC axes reflect directions of covariability enriched in post-task. Because these directions can distinguish spatially related firing during the task, this shows the enrichment of certain spatial information in the post-task. The interpretation of the result as "replay" should be better unpacked.

We thank the reviewer for these suggestions, and we have updated Fig. 3 to include them. The suggestion to include the results from PCA on the pre-task, task, and post-task data was particularly helpful. This analysis found two things: 1) the gcPCs look similar to the task PCs, which is suggestive of replay, and 2) the pre- and post-task PCs look similar to each other, but not the task PCs or gcPCs, suggesting that pre- and post-task PCA are extracting shared sources of variance that are not related to the task.

What is the significance that start and air puff are overlapping in the danger run (top left of Fig. 3D), but separated in the safe run (bottom right of Fig. 3D)? What is the significance of the segregation of the blue (pre air-puff) and red lines (post air-puff)? Why are the start, non-airpuff and stop in the safe run separated in both pre-task and post-task subspaces?

We have revised Fig. 3 to clarify the main point of relevance to this study, namely that gcPCA on the post/pre-task data yields structure similar to PCA on the task data itself, which is suggestive of replay. We do not know exactly why the trajectories look the way they do. This is an

interesting question about the neurophysiology of the hippocampus that we think is beyond the scope of this technical paper.

- In Figure 3 the authors mention they are using non-orthogonal gcPCA but they show the trajectories plotted against orthogonal axes. I assume that the authors are just orthogonalizing the pairs of dimensions, but it would be good to be explicit about this. In fact, it would be interesting to plot the non-orthogonal axes as arrows (i.e. point-to-tip visualization of a vector) in the low-D plots of Figure 3, or as a supplemental figure.

We apologize for the lack of clarity in the original manuscript. The question of orthogonality is more complicated than we made it out to be, and we have revised the manuscript to clarify this issue. A useful interpretation of how gcPCA works is that it first applies a whitening transformation to the data to penalize high-variance dimensions. It then applies standard PCA or cPCA in the whitened space and projects back out into the original feature space. To clarify what we said before, standard gcPCs actually are orthogonal, but they are orthogonal in the whitened feature space, not the original feature space. For this reason, the main plots in the paper use orthogonal axes. In practice, we have found that the difference between standard and orthogonal gcPCA is very small and likely irrelevant in most cases. This is a subtle point that we address with the new Fig. S3, as well as in the text (lines 206-208, 392-393, 467-472).

- The results on Figure 4 seem similarly short on details. However, I am not familiar with the scientific literature on ‘omics analysis to give detailed comments on this part of the manuscript

In the revised Fig. 4 we include additional analyses including the controls requested by the reviewer for the replay analysis in Fig. 3. The new Fig. 4 is much clearer because it illustrates how and why PCA and gcPCA yield different results. Specifically, PCA tends to extract features that are shared between the two datasets, but gcPCA extracts features that differ between them.

An additional change is that the revised Figure 4 now uses the sparse version of gcPCA. We realized the original manuscript did not illustrate the use of sparse gcPCA, and identifying genes of interest is a good application for it. We have also updated and improved the implementation of sparse gcPCA. The new version corrects an error in how the penalty terms were calculated and is substantially more efficient computationally.

Reviewer #2: In this paper, the authors propose a variant of contrastive PCA, an recently-developed algorithm that identifies differing patterns between two contrasted datasets. The authors' contribution is a set of algorithms that are parameter-free unlike the original algorithm and also more intuition into the outputs of the original algorithm its limitations and suggestions on how to overcome these. Application of these algorithms to three datasets showcases their features well. I found the paper informative, insightful, and well-written and have a few suggestions that could strengthen its impact and usability:

a) I found the distinction that the authors provide between discriminant analyses and cPCA very useful and intuitive for prospective users of the methodology. I wonder if the authors could also

apply LDA-like analyses to one of the datasets they used here and compare/contrast the results with gcPCA to offer more intuition into these differences. This would be very informative for the readers to appreciate the usefulness of cPCA and gcPCA in particular here and understand when these are more suitable for their analyses over LDA.

We appreciate the reviewer's suggestion, and we now include Fig. S1, which has a diagram illustrating the difference between gcPCA and LDA, as well as a comparison of gcPCA vs. LDA on the face dataset in Fig. 2. We also included an LDA panel in Fig. 4.

b) Can we get some more intuition into what constitutes "infinite data" in this context? Is there a threshold that the authors have empirically determined? Or are all actual datasets finite?

This is a good question, but unfortunately it is complicated, and we do not have a rigorous answer. The answer would differ depending not only on the dimensionality and covariance structure of the data in the limit of large N, but also on higher order statistical properties that influence the sampling error for estimates of the covariance matrix. In real-world experimental data there are also other factors, such as measurement noise and deviations from stationarity, that would further increase the amount of data required. In practice, we advocate for treating all real-world datasets as finite, particularly in cases like this when there is little downside in doing so. We have added a brief section in the discussion (lines 405-407) discussing this.

c) Some more intuition into when orthogonality or sparsity would be useful in this context would help too. For example, what outputs would the orthogonal or sparse gcPCAs give for one of the examined datasets? How would these differ from the unconstrained ones? Are there other caveats in including such constraints, e.g. algorithm convergence or complexity?

We thank the reviewer for this helpful suggestion, which we address in the new Fig. S3. Fig. S3 compares the results from analyzing the hippocampal data using standard, orthogonal, and sparse gcPCA. For this dataset, the orthogonality constraint yields results that are almost identical to standard gcPCA, but the sparsity constraint results in a more substantial difference. Standard gcPCA is the fastest to compute, and orthogonal gcPCA and sparse gcPCA are slower. Regarding convergence, sparse gcPCA uses gradient descent, but it is a convex problem, and in practice we have not observed any problems. We have added text explaining these points (lines 299, 398-400, 542-543).
